# A review of older adults’ acceptance of health monitoring tools: application status, theoretical acceptance models, and user-related and context-level influencing factors

**DOI:** 10.3389/fpubh.2026.1937187

**Published:** 2026-10-15

**Authors:** Qi Zhang, Yongqin Zhang

**Affiliations:** School of Nursing, Henan University of Chinese Medicine, Zhengzhou, China

**Keywords:** acceptance, health monitoring, health monitoring tools, influencing factors, older adults

## Abstract

Health monitoring tools enable real-time, accurate monitoring of physiological data in older adults, offering timely alerts and feedback that facilitate early detection of health problems and prompt intervention. However, adoption and use of these tools among older adults remains limited, significantly constraining their practical effectiveness. This narrative review examines the factors influencing older adults’ acceptance of health monitoring tools by synthesizing 49 studies, comprising 40 studies classified as core evidence and 9 as supplementary contextual evidence. Relevant literature was identified through a snowballing approach, beginning with key articles and tracing their reference lists and citing articles. Findings were synthesized through a thematic approach. Among the theoretical models identified in this review, the Technology Acceptance Model and the Unified Theory of Acceptance and Use of Technology are the most frequently applied, whereas the Senior Technology Acceptance Model, despite its specific relevance, has not been applied to health monitoring tool acceptance in any study included in this review. Key influencing factors can be grouped into two categories: user-related factors, such as demographic, health-related, and psychological characteristics; and tool- and context-level factors, such as privacy and security of monitoring data, interaction experience and ease of use, device appearance and invasiveness, and economic costs associated with pricing and technology access. Enhancing acceptance may require a comprehensive approach encompassing targeted health education and digital skills training, optimized user interfaces and privacy safeguards, multi-tiered financial subsidies, and the development of innovative theoretical models tailored to the older adult population.

## 1 Introduction

Global population aging is advancing at an unprecedented pace. According to 2024 World Health Organization data, by the mid-2030s, the population aged 80 years and older is projected to reach 265 million, surpassing the number of infants under one year of age; by the end of the 2070s, the population aged 65 years and older is expected to reach 2.2 billion (1). The aging trend is particularly pronounced in some countries. For example, in China, the proportion of older adults is projected to exceed 30% by 2035, marking its transition to a severely aging society (2, 3), while many other low- and middle-income countries are also experiencing rapid aging.

The accelerating population aging and the growing burden of chronic diseases among older adults have made health management for older adults a major concern across all sectors of society (4). The continuous advancement of technologies such as artificial intelligence (AI) and Internet Plus has led to the increasingly widespread application of health monitoring tools in the early detection, early diagnosis, and early treatment of chronic diseases among older adults (5). Health monitoring tools, as novel technologies and approaches in modern medical science, can assist older adults in chronic disease management and self-monitoring of health status, which may reduce the clinical burden on healthcare providers (6–8), and hold profound significance for improving the health outcomes of the older adults and optimizing the allocation of healthcare resources.

The concept of “health monitoring” first originated in the United States, referring to the provision of real-time physiological information and scientifically effective intervention recommendations for populations through data analysis, assessment, and prediction (9). Health monitoring tools are personal-level devices simplified from large-scale professional medical equipment; they can monitor and collect health data of older adults in real time during daily life, assist them in scientifically managing chronic diseases and preventing acute illnesses, and have been widely applied in the geriatric industry (10). In response to the diverse needs of older users, families, communities, and institutions, a variety of health monitoring tools have been introduced in different countries. Currently, commonly used health monitoring tools include wearable devices [e.g., smart bands, smartwatches, skin patches, and smart vests (11–14)], smart wheelchairs (15), smart canes (16), smart home systems (17), and other monitoring systems (18). These tools vary widely in form and function, and their acceptance drivers may differ across types. This review considers health monitoring tools as a broad category, while noting technology-specific findings where relevant. Through real-time physiological data acquisition, health analysis, and early warning functions, these tools provide health management services for older adults.

Research shows that older adults have a relatively high willingness to use health monitoring and safety surveillance tools (19); however, actual use remains far from encouraging. A 2024 survey of older adults—who were on average 73.75 years old, predominantly female (79%), mostly Black or African American (58%), and the vast majority of whom (91.6%) had completed high school or higher education—showed that 95.8% of older adults had never used a smartwatch (20). Another study conducted in 2023 of 400 community-dwelling older adults in Hangzhou—who were predominantly aged 60–75 years, with balanced gender distribution, moderate health status, and mostly urban residency—found that the utilization rate of wearable devices was 29.7% (21). Meanwhile, in a 2023 study among 98 older exercisers in Xiamen who had used wearable devices, the discontinuation rate reached 53.1%, with the primary reasons including perceived lack of practicality and operational complexity (22). A 2023 web-based survey of 174 older adults aged ≥65 years—who were predominantly aged 65–75 years, nearly balanced in gender, and with a relatively high education level (68.9% having undergraduate or graduate education)—reported that only 23% of older adults knew about or had used smart home devices (17). These data were collected in different years and drawn from older adult populations across regions with different levels of economic development, age groups, and living arrangements. The variation in sample characteristics and economic contexts may partly account for the considerable discrepancies observed across study findings. There is a notable gap between older adults’ high willingness to use health monitoring tools and their low actual adoption. Research has shown that some older adults have low adoption of these tools, which may compromise their effective utilization (23, 24). The reasons for this gap are multifaceted: on the one hand, limitations in technological maturity, ease of use, and portability mean that tools such as smart vests, smart wheelchairs, and smart canes with health monitoring functions are still in the early stages of development and market validation (25); on the other hand, factors such as product cost and price, the economic status of older adults, their lifestyle habits, and health consumption attitudes also exert an influence (26). To date, research in this field has predominantly focused on technology development and usability testing of individual tools, and a systematic exploration of the factors influencing older adults’ acceptance remains insufficient (27–29).

Despite the current low adoption rates, the anticipated prospects for health monitoring tools remain positive, and related research is growing rapidly. Garcia-Moreno et al. (30) used smartwatches to monitor vital signs and activities of daily living in older adults and analyzed their frailty risk based on daily activities, which may simplify the diagnosis of frailty syndrome and help older adults maintain independence and improve their quality of life. Wu et al. (31) employed smartwatches to continuously monitor oxygen saturation and guide breathing exercises in older adults with chronic obstructive pulmonary disease (COPD), helping them develop self-care skills and modify their lifestyle, which may enhance the overall effectiveness of disease self-management. Thurston et al. (32) further addressed Alzheimer’s disease risk prediction by using ambulatory skin conductance monitoring to detect nocturnal vasomotor symptoms in menopausal women and provide timely early intervention. In addition, Della Monica et al. (33) applied smart wristbands to monitor sleep–wake rhythms in older adults with dementia, recording physiological data and polysomnography during sleep; this not only accurately identified nighttime sleep apnea symptoms but also demonstrated the great potential of health monitoring tools for long-term home use. Siva et al. (34) and Ng et al. (35) applied radar sensors and home monitoring to fall risk detection in older adults. By monitoring and analyzing gait characteristics and balance ability, they facilitated timely intervention after a fall was detected, which may reduce adverse outcomes from falls and effectively improve the quality of life of older adults.

Acceptance is an important indicator of the extent to which individuals or groups embrace new things and new technologies (36). The acceptance of health monitoring tools among older adults reflects their willingness and attitudes toward using such tools for health management, and it is not only associated with the effectiveness of health management in this population but also may influence the dissemination and acceptance of these tools (37, 38). Studies have demonstrated that older adults’ acceptance of health monitoring tools is affected by multiple factors (39–41). However, studies examining the factors influencing acceptance among older adults across diverse cultural contexts remain relatively scarce, particularly in low- and middle-income countries. Exploring the acceptance of health monitoring tools among older adults and its influencing factors is therefore of guiding significance for promoting the application of such tools. Health monitoring tools are still at an exploratory and developmental stage. Although a growing number of primary studies have examined older adults’ acceptance of these tools, the evidence remains limited and fragmented.

Consequently, this review aims to synthesize the fragmented evidence on factors influencing older adults’ acceptance of health monitoring tools, in order to clarify the current state of research and provide useful insights for future research and practice.

## 2 Methods

This is a narrative review. Given the heterogeneity of the literature and the exploratory stage of research on older adults’ acceptance of health monitoring tools, a narrative approach was adopted to synthesize theoretical models and influencing factors.

Relevant literature was identified through a snowballing approach (42). A key seed article was Dai and Lin (9), which examined home-dwelling older adults’ acceptance of health monitoring products using an extended UTAUT model. Its reference list and citing articles were used as starting points for further literature identification. Both backward and forward citation tracking were performed. Backward snowballing involved screening the reference lists of seed articles. Forward snowballing involved identifying studies that cited the seed articles, using Web of Science, PubMed, and CNKI. The process was iterative: newly identified relevant articles were used as new seed articles, and their reference lists and citing articles were also screened. The process continued until further searching yielded no new relevant studies or themes. These databases were used only for citation tracking and full-text retrieval, not for a systematic database search with predefined search strings.

Studies were considered relevant if they addressed the acceptance, adoption, use, or continuance of health monitoring tools among older adults, or if they reported factors influencing these outcomes. Both English and Chinese publications were included. No restriction was placed on study design. Only primary studies were included; reviews were excluded to avoid double-counting of evidence. Older adults were the core population of interest. Studies were further categorized into core evidence (Table 1) and supplementary contextual evidence (Table 2). Core evidence included studies directly involving older adults and evaluating acceptance, behavioral intention, adoption, use, continuance, or their determinants. Supplementary contextual evidence included simulation studies of older adults, studies of family caregivers of older adults, or studies addressing relevant technical or design issues. These studies were retained only when their findings contributed directly to understanding older adults’ acceptance.

**Table 1:** Characteristics of core evidence.

Author	Country	Sample	Design	Technology type	Theoretical framework	Key findings
Dai and Lin (9)	China	161 home-dwelling older adults aged 55–80 years	Cross-sectional survey; structural equation modeling (SEM)	Health monitoring products	Extended UTAUT model	Effort expectancy, social influence, perceived value, and product features influenced behavioral intention; performance expectancy and perceived risk were not significant; experience, education, and self-rated health status had no significant moderating effects.
Grammes et al. (13)	Germany	6,849 insulin-treated older adults with diabetes aged ≥60 years	Prospective study	Continuous glucose monitoring (CGM)	–	CGM use increased over time; younger age was a main predictor; CSII use in type 1, and longer diabetes duration, higher BMI, and CSII use in type 2 were also associated with use; age-related barriers, insufficient usability, and lack of interest may affect use.
Lin et al. (14)	Taiwan, China	12 older adults and their family caregivers	Qualitative study	Smart vest with remote monitoring system	TAM	Incompatibility with the home environment, data transmission interruptions, and inaccurate data sources were barriers.
Esmaeilzadeh. (17)	USA	174 older adults aged ≥65 years	Qualitative study; web-based interview survey; content analysis	Intelligent toilet seats	–	Five themes emerged: health-related benefits, technology-related advantages, and use-related benefits; concerns included psychological concerns and technical performance risks.
Tian and He (19)	China (Xinjiang)	538 older adults aged ≥60 years	Cross-sectional questionnaire survey	Health monitoring tools	Fishbein model (Theory of Reasoned Action)	Health monitoring, safety monitoring, and leisure/entertainment were most demanded; behavioral attitude and subjective norms influenced consumption.
Li et al. (20)	USA	24 older adults (mean age 73.75 years)	Qualitative study; semistructured interviews; directed content analysis	Wrist-worn smartwatches	UTAUT2	Six themes emerged; first-time users reported high acceptance; challenges included battery, accuracy, syncing, and showering; devices increased motivation and activity.
Luo et al. (21)	China (Hangzhou)	400 community-dwelling older adults	Cross-sectional questionnaire survey	Smart medical care	–	Awareness and utilization were low; home monitoring devices had the highest willingness; barriers included policy, age-friendly design, and caregiver dependence.
Lin et al. (22)	China (Xiamen)	98 older exercisers aged ≥60 years	Cross-sectional questionnaire survey	Wearable devices	–	Awareness was high but usage was low, with high discontinuation; reasons included lack of practicality, complexity, privacy concerns, discomfort, and poor battery; usage was positively associated with education and income, and negatively with age.
Teh et al. (29)	Malaysia	30 older adults aged 60–78 years	Qualitative study; in-depth interviews; inductive thematic analysis	Digital health coaching app (AGATHA)	–	Six themes emerged; the app was perceived as accessible, engaging, and beneficial for health literacy and digital skills; suggestions included more interactivity, personalization, and visual appeal.
Wang et al. (37)	China	193 older adult patients	Cross-sectional questionnaire survey	Mobile health (mobile medical services)	–	Willingness was low; influencing factors were innovation/convenience and personal factors; main barriers were traditional care-seeking habits and lack of awareness.
Claes et al. (38)	Belgium	245 adults aged ≥60 years	Cross-sectional survey; validated questionnaire	Contactless in-home monitoring sensors	–	Older adults found contactless monitoring useful; were willing to share data and participate in monitoring decisions; concerns included functioning and costs; acceptance was influenced by sociodemographic factors and attitudes/perceptions.
Zhou et al. (40)	China (Yangtze River Delta)	200 older adults aged ≥60 years with smart home experience	Cross-sectional questionnaire survey; structural equation modeling	Smart home devices	Extended TAM	Perceived usefulness and perceived ease of use positively influenced intention to use; intergenerational technology support, perceived value, and perceived risk influenced perceived usefulness and perceived ease of use.
Liu and Qiao (45)	China	4 older adults using intelligent walking aids	Mixed-method design; structural equation modeling	Intelligent walking aids	STS-TAM integrated model	Product design elements (intelligence, function, appearance) affected social perceptions; perceived social psychological inclusion influenced perceived usefulness and social activity; social activity and perceived usefulness influenced behavioral intention.
Liu and Xu (46)	China	204 older adults aged ≥60 years	Quantitative questionnaire survey; structural equation modeling	Intelligent health monitoring APP	Extended TAM	Perceived usefulness had the strongest influence; perceived ease of use was influenced by the interface; intention was influenced by usefulness, ease of use, security, interface, and enjoyment
Wu and Lim (47)	China	389 older adults aged ≥60 years	Cross-sectional questionnaire survey; structural equation modeling	Smart wearable devices	UTAUT2 integrated with Technology Readiness Index (TRI)	Performance expectancy, effort expectancy, social influence, facilitating conditions, hedonic motivation, price value, and digital health literacy positively influenced intention; digital health literacy negatively moderated the expectancy–intention relationship.
Tian and Wu (48)	China	1,453 older adults aged ≥60 years with chronic diseases and mHealth experience	Cross-sectional questionnaire survey; structural equation modeling	Mobile health	Integrated ECM-ISC and UTAUT model	Confirmation influenced satisfaction and expectancies; satisfaction, performance expectancy, effort expectancy, social influence, and facilitating conditions influenced continuance intention; performance expectancy had the strongest effect.
Chen and Chan (50)	Hong Kong, China	1,012 older adults aged ≥55 years	Cross-sectional questionnaire survey; empirical testing of STAM	Gerontechnology	Senior Technology Acceptance Model (STAM)	STAM was supported; individual attributes, health and ability characteristics, and facilitating conditions directly affected acceptance, and were better predictors than usefulness and ease of use.
Camp et al. (51)	UK	33 community-dwelling older adults	Qualitative study; semistructured interviews with photo-elicitation	In-home monitoring technologies	Framework by Peek et al.	The older group was more accepting of wearable sensors, while the younger group preferred environmental sensors; overall acceptable, with concerns about privacy, cost, reduced human contact, and data security.
Cavallo et al. (52)	Italy	45 older adults aged 65–86 years	Experimental study; questionnaires	Robotic services (Robot-Era system: DORO, CORO, ORO)	–	Participants had a positive impression of the robots; the system was perceived as socially acceptable and useful for supporting independent living; higher education and male participants gave higher scores.
Deguchi et al. (53)	Japan	899 older adults aged ≥65 years	Cross-sectional study	Smartwatches	–	Acceptance was associated with younger age and health factors in men, and with younger age, living alone, income, and digital literacy in women.
Simmich et al. (54)	Australia	19 older adults with COPD	Qualitative study; semistructured interviews; content analysis	Wearable activity trackers and active video games	Social Cognitive Theory (SCT)	Wearable activity trackers helped with activity quantification, goal-setting, and reminders, and participants were willing to share data with clinicians; game motivators were fun, social interaction, and health benefits, while barriers were difficulty, competition, equipment, and embarrassment.
Kadylak and Cotten (55)	USA	1,148 older adults aged ≥65 years	Cross-sectional online survey	Emerging technologies including health monitoring tools	–	Willingness to use emerging technologies was generally low and varied by technology type; health-related variables were associated with willingness to use some technologies.
Doyle et al. (56)	Ireland and Belgium	120 older adults with multimorbidity	Semistructured interviews; validated questionnaires	Digital platform with sensors	–	Most continued to use; usable and low burden; benefits were improved self-management, health, and well-being; barriers were poor health and device issues.
Abdul Rahman et al. (58)	Malaysia	336 community-dwelling older adults aged ≥50 years	Cross-sectional survey	Fall detection devices	–	Most older adults were interested in fall detection devices and expected them to be user-friendly, affordable, and accurate.
Avella-Rodriguez et al. (59)	Colombia	6 older adults aged ≥65 years, caregivers, and healthcare professionals	Mixed-methods study; semistructured interviews and online surveys; thematic analysis	Wearable fall detection systems	–	Stakeholders viewed the systems as useful and non-stigmatizing, but had privacy concerns; they preferred small, lightweight devices that can send help messages.
Street et al. (60)	Australia	84 older adults aged ≥55 years living independently	Qualitative study	Smart technologies	–	Older adults showed cautious and conditional acceptance; benefits included convenience, safety, and independence; concerns included isolation, privacy, surveillance, and stigmatization; technologies should augment rather than replace human care.
Kim and Choi. (62)	South Korea	170 older adults	Cross-sectional survey; face-to-face interviews	Healthcare technology and services	TAM; five dimensions of privacy concern	Older adults were selective about sharing, preferring family and hospitals; education, age, technology attitudes, gender, and self-rated health influenced willingness.
Kim et al. (63)	South Korea	27 community-dwelling older adults with chronic diseases	Quantitative descriptive statistics and qualitative content analysis	Mobile health (mHealth)	eHealth Enhanced Chronic Care Model (eCCM)	Most completed; learning and confidence were affected by demographic factors; interactive and personalized approaches were preferred; technical challenges were common; family support and tailored training were important.
Portz et al. (65)	USA	24 older adults with multiple chronic conditions	Qualitative descriptive study	Patient portal	TAM	The portal was perceived as easy to use and useful; intention was influenced by ease of use, usefulness, prior negative experiences, and lack of awareness; challenges included log-in and interface design.
Frishammar et al. (66)	Sweden	Older adults	Qualitative study	Digital health platforms	–	Barriers to initial adoption were negative attitudes and technology anxiety; lack of trust affected both adoption and usage; suggestions included app improvements and tailored education.
Zhang et al. (67)	China	35 older adult users of digital medical apps	Qualitative study using grounded theory	Digital medical apps	Human-Information Processing	Built a four-dimension evaluation system; information design was highest priority; key strategies were reducing information density, simplifying interface, enhancing feedback, and shortening paths.
Wu et al. (68)	France	11 older adults (mean age 79.3 years)	Mixed-method study	Assistive robot (Kompaï)	Adapted UTAUT model	Intention was low and attitudes were negative; seen as easy and amusing but not useful; direct experience did not change acceptance; barriers were uneasiness, stigmatization, and ethical concerns.
Cajita et al. (70)	USA	10 older adults with heart failure	Qualitative descriptive exploratory study; semistructured interviews	Mobile health (mHealth)	TAM	Facilitators were technology experience, ease of use, and doctor’s recommendation; barriers were lack of knowledge, poor interface, and cost; older adults were willing but with reservations.
Tanaka et al. (71)	Japan	21 older adult-related participants	Qualitative study	Wearable technology for home health monitoring	–	Most older adults were interested in home monitoring devices, especially if not complicated; healthcare workers found them useful during COVID-19; cost and technical issues were barriers.
Wong et al. (72)	Singapore	27 community-dwelling older adults	Qualitative descriptive study; three sessions of semistructured interviews; thematic analysis	AI-based health technologies	Theoretical Domains Framework and COM-B model	Participants acknowledged potential benefits of AI but emphasized the irreplaceable role of human expertise; concerns included usability, privacy, and security; key strategy was integrating AI with healthcare providers.
Fiorini et al. (73)	Italy	78 community-dwelling older adults	Longitudinal real-world pilot study (12 months); linear mixed-effects models; k-means clustering	Assistive technologies (socialization platform and health/environmental monitoring system)	Almere Model; System Usability Scale (SUS)	Two baseline profiles were identified; the digitally resilient profile had higher usability and acceptance; technostress and loneliness were consistent negative predictors.
Zhang et al. (74)	China (Nanjing)	16 older adults with chronic diseases and 11 healthcare providers	Qualitative descriptive study; semistructured interviews	Wearable devices for home health monitoring	TAM and Health Belief Model (HBM)	Benefits were widely recognized; complexity, cost, and data security were key obstacles.
Cimperman et al. (75)	Slovenia	400 older adults aged ≥50 years	Cross-sectional survey; structural equation modeling	Home Telehealth Services (HTS)	Extended UTAUT model	Performance expectancy, effort expectancy, facilitating conditions, and perceived security influenced intention; computer anxiety negatively influenced effort expectancy; social influence was not significant.
Wong et al. (77)	Hong Kong, China	76 community-dwelling older adults	Feasibility randomized controlled trial; 3-month intervention; follow-up at baseline, 1, 3, and 6 months	Wearable monitoring device	Self-Determination Theory (SDT); usability questionnaire adapted from Senior TAM	No significant differences in continued use intention, quality of life, or health service utilization; ongoing support may promote sustained use.
Paolillo et al. (79)	USA	175 community-dwelling older adults	Observational study; questionnaires	Wearable activity tracker (Fitbit Flex 2)	–	Wearing and syncing adherence were high; wearing adherence was related to female sex, and syncing adherence was related to memory; participants were generally willing to participate again.

**Table 2:** Characteristics of supplementary contextual evidence.

Author	Country	Sample	Design	Technology type	Theoretical framework	Key findings
Wang et al. (11)	Canada	28 university students simulating older adults	Experimental study	Contactless smart home sensors	–	Random forest performed best; environmental factors were most important
Wu et al. (31)	Canada	34 older adults with COPD	Prospective before-after cohort feasibility study	Smartphone and smartwatch self-management application	–	Older adults with COPD used the app, but no significant improvements were found in self-management, quality of life, disease control, or self-efficacy.
Wang et al. (49)	China	406 healthcare wearable device users	Cross-sectional questionnaire survey; partial least squares structural equation modeling	Healthcare wearable devices	Integrated model of UTAUT and TTF	Performance expectancy, effort expectancy, facilitating conditions, social influence, and task-technology fit positively influenced intention; task and technology characteristics indirectly influenced intention through task-technology fit and effort expectancy.
Herkert et al. (57)	Netherlands	10 older adults with advanced combined chronic cardiac and pulmonary disease	Single-center feasibility study	Wrist-worn activity tracker rehabilitation device	–	Most completed the program; adherence declined from early to late phase; high satisfaction; some technical issues; PSC scores improved; no adverse events.
Comiskey et al. (61)	Ireland	14 informal carers of older adults with long-term conditions	Descriptive qualitative study; interviews and focus group; thematic analysis	Video-based home monitoring system (BREATHE)	–	Carers conditionally accepted video monitoring for emergencies; acceptance in non-emergencies was mixed.
Vanoh et al. (64)	Malaysia	73 participants, including 30 older adults, caregivers, health professionals, and IT experts	Mixed-methods; development and acceptance study of a web-based intervention	Web-based education tool (WESIHAT 2.0) for healthy cognition	–	Older adults and caregivers were generally satisfied; suggestions included larger font, shorter sentences, more images, and avoiding unfamiliar terminology.
Dermody et al. (69)	USA	10 family caregivers of care-dependent older adults	Qualitative descriptive study; semistructured interviews; thematic analysis	Health smart home technology	–	Five themes influenced caregiver readiness (big brother effect, framing for acceptance, data privacy, burden, cost); caregivers were open, but readiness depended on older adults; main concern was overcoming older adults’ hesitancy.
Nascimento et al. (76)	Canada	55 older adults	Observational study; 2 experiments	Wearable mobility monitoring devices	–	ActiGraph wGT3X-BT was most feasible; thigh-worn device detected sedentary behavior and ankle-worn device provided step counts and posture; willingness to wear was high.
Guo et al. (78)	China	21,881 older adults	Quantitative secondary analysis; bidirectional fixed-effect models; mediation analysis	Smart wearable devices	–	Smart wearable device use improved physical health; the effect was stronger in older, those without chronic diseases, and those living with children; exercise frequency and duration were mediating pathways.

Two reviewers independently screened titles and abstracts. Both had a background in geriatric nursing and older adult health management. Disagreements were resolved by discussion. Full texts of potentially relevant articles were then reviewed. Data were extracted on author, year, country, sample, design, technology type, theoretical framework, and key findings. Studies were then categorized as core evidence or supplementary contextual evidence based on their population and outcome focus.

Findings were synthesized thematically (43). Categories of influencing factors were inductively derived from recurring themes in the literature, with reference to existing technology acceptance models (44). No formal quality assessment was conducted because the included studies were highly heterogeneous in design, sample, and outcome measures, and the review aimed to synthesize theoretical models and influencing factors rather than to grade evidence quality. No quantitative synthesis was performed, consistent with the narrative nature of this review.

Based on the thematic synthesis, the following operational definitions and classification were developed. Acceptance is used as the core concept, referring broadly to older adults’ attitudes and willingness toward health monitoring tools. Adoption refers to the initial decision to use a tool, use refers to actual usage behavior, and continuance refers to sustained use over time. Where source studies reported these outcomes, their original terminology is retained because these concepts may have different determinants. User-related factors refer to characteristics of older adults themselves that may shape their acceptance of health monitoring tools, including demographic characteristics (age, gender, educational attainment), health-related characteristics (health status, multimorbidity, functional ability), and psychological characteristics (anxiety, technology confidence, concerns about stigmatization). Tool- and context-level factors refer to attributes of the tools and the broader environment in which they are used, including interaction experience and ease of use, device appearance and invasiveness, privacy and security, and economic cost. This classification was adopted because factors at these two levels may require different intervention strategies: user-related factors are primarily addressed through education, training, and psychological support, whereas tool- and context-level factors are primarily addressed through design optimization, privacy safeguards, and policy interventions.

## 3 Results

This review presents its findings through a thematic synthesis, organized into theoretical models of acceptance and factors influencing older adults’ acceptance of health monitoring tools. The latter are grouped into user-related factors (demographic, health-related, and psychological characteristics) and tool- and context-level factors (interaction experience, device attributes, privacy, security, and economic cost).

The 49 studies (9, 11, 13, 14, 17, 19–22, 29, 31, 37, 38, 40, 45–79) reviewed covered wearable devices, smart home systems, mobile health applications, sensors, and assistive robots. Of these, 40 studies were classified as core evidence and 9 as supplementary contextual evidence. The characteristics of these two groups are presented in Tables 1 and 2, respectively. Geographically, 25 studies were conducted in Asia (17 in China, 3 in Malaysia, 2 each in Japan and South Korea, and 1 in Singapore), 11 in Europe (2 each in Italy, Ireland, and Belgium, including one cross-national study conducted in Ireland and Belgium; 1 each in Germany, the United Kingdom, the Netherlands, Sweden, France, and Slovenia), 10 in North America (7 in the United States and 3 in Canada), 2 in Oceania (Australia), and 1 in South America (Colombia). Study designs included cross-sectional surveys, qualitative interviews, mixed-methods studies, experimental studies, and longitudinal pilots. The Technology Acceptance Model (TAM), the Unified Theory of Acceptance and Use of Technology (UTAUT), and their extensions were the most frequently used theoretical models among the studies identified in this review.

### 3.1 Theoretical models of acceptance of health monitoring tools

Theoretical models of acceptance explain and predict people’s acceptance of new technologies and tools by theoretically deriving the relationships among various variables. These models are associated with factors such as users’ subjective cognitive beliefs, the characteristics of the technology itself, and the context of application. Scholars worldwide have extensively investigated the factors influencing the acceptance of health monitoring tools from different perspectives. They have extended and refined existing technology models, constructed new theoretical models, and integrated multiple theoretical models to analyze the influencing factors, with the aim of enhancing the specificity of interventions. A number of studies indicate that when developing and promoting health monitoring tools, it is essential not only to consider the needs of older adults but also to conduct analyses based on various theoretical models. This holds important guiding significance for improving older adults’ acceptance of these tools. In addition, the Senior Technology Acceptance Model (STAM) has been shown to be effective in research on technology acceptance among older adults. Chen and Chan (50) validated STAM in the context of gerontechnology. However, no study included in this review has applied STAM to the acceptance of health monitoring tools. The application of major theoretical models of acceptance is summarized in Table 3.

**Table 3:** Theoretical models of acceptance and their applications.

Model references	Description	Strengths	Limitations	Applications in health monitoring tools
TAM (112)	The model is widely used to explore consumers’ acceptance of emerging innovations and the key variables influencing acceptance, encompassing two core constructs: perceived usefulness and perceived ease of use. It was later extended and revised, leading to the development of TAM2 (113) and TAM3 (114).	Strong generalizability; the most frequently employed model in empirical acceptance research (115).	Susceptible to interference from subjective factors; limited explanation of external variables and social influences; lack of specificity and limitations when applied to particular contexts (45, 80).	① Smart home systems (40): Perceived usefulness and perceived ease of use positively influenced older adults’ intention to use smart home systems, and perceived ease of use exerted a positive effect on perceived usefulness.
				② Wearable medical apps (46, 116): The interface is influenced by perceived ease of use, while perceived usefulness, perceived security, and perceived enjoyment are influenced by the interface.
				③ Wearable device monitoring systems (117): A device usage feedback form was designed based on TAM. The results indicated that the design of wearable devices in hospital wards should be centered on nurses’ operational needs and take environmental suitability into consideration.
UTAUT (118)	This model is widely used to explain or predict the underlying factors that determine individuals’ acceptance or rejection of a particular technological behavior. It comprises four key constructs: performance expectancy, effort expectancy, social influence, and facilitating conditions. Three additional dimensions—hedonic motivation, price value, and habit—were later incorporated, leading to the development of UTAUT2 (119).	Relatively strong explanatory and predictive power in technology acceptance research; broad scope of application.	Involvement of multiple variables makes model parameters complex; challenges in both understanding and application.	① Health monitoring products (9): By incorporating new variables into UTAUT, a hypothetical acceptance model for health monitoring products targeting home-dwelling older adults was constructed. The results revealed that effort expectancy and social influence from UTAUT significantly influenced older adults’ acceptance of health monitoring products.
				② Wearable devices (47): By integrating UTAUT2 with the technology readiness index, it was identified that performance expectancy, effort expectancy, social influence, facilitating conditions, hedonic motivation, price value, and digital health literacy are positive predictors of older adults’ intention to use smart wearable devices.
ECM-ISC (120)	This model was developed by integrating Expectation-Confirmation Theory with TAM. It encompasses core constructs including expectation confirmation, perceived usefulness, satisfaction, and continuance intention, and is widely applied to investigate users’ continuance intention in information systems.	Ability to reflect the actual process underlying users’ continuance intention.	Assessment of the satisfaction dimension lacks objective criteria and still requires further refinement (81).	① Mobile health (48): UTAUT was incorporated into ECM-ISC to explore satisfaction and continuance intention regarding mobile health. The results indicated that effort expectancy, performance expectancy, social influence, and facilitating conditions were the main influencing factors.
TTF (121)	This model was developed to address the limitations of TAM in matching systems to specific tasks, and is applied to examine the relationships between technology characteristics, task characteristics, and their effects on performance and adoption rates.	Strong extensibility, which facilitates integrative research with other models (82).	Data on technology and task characteristics are not easily obtainable, and the model parameters are relatively difficult to interpret.	① Wearable devices (49): By integrating UTAUT and TTF model, the results indicated that consumers’ acceptance of medical wearable devices is positively influenced by user perception and task-technology fit.
STAM (50)	This model was specifically designed for the older adult population by scholars in Hong Kong, China. Built upon TAM and UTAUT, it incorporates age-related health and capability factors to assess older adults’ acceptance of gerontechnology (electronic or digital products or services that can enhance older adults’ independent living and social participation, enabling them to remain relatively healthy, comfortable, and safe).	Unique applicability and substantial significance in research on technology acceptance among older adults. Strong specificity, rich set of variables, and alignment with the characteristics of older adults.	Pays insufficient attention to product design optimization and the needs of older adults (100).	Validated in gerontechnology; not applied to health monitoring tools in the included studies.

TAM, Technology Acceptance Model; UTAUT, Unified Theory of Acceptance and Use of Technology; ECM-ISC, Expectation-Confirmation Model of IS Continuance; TTF, Task-Technology Fit; STAM, Senior Technology Acceptance Model.

Among the five models reviewed, TAM and UTAUT are the most frequently applied in the studies identified in this review, with the latter offering relatively strong explanatory power due to its incorporation of a broader set of variables (45, 80). The Expectation-Confirmation Model of IS Continuance (ECM-ISC) has been introduced more recently and is primarily used to examine sustained use rather than initial adoption (81). The Task-Technology Fit (TTF) model, with its strong extensibility, is increasingly used in combination with TAM or UTAUT, which may enhance predictive power (82). STAM, despite its specific relevance to older adults, has not yet been applied to health monitoring tools in the included studies.

### 3.2 Factors influencing older adults’ acceptance of health monitoring tools

The health monitoring tools reviewed varied in form and function, including wearable devices (e.g., smartwatches, wristbands, skin patches, smart vests), smart home systems, ambient and radar sensors, mobile health applications, and assistive robots. These tool types differ in several respects: wearable devices require active user involvement and are highly visible, smart home and ambient systems operate passively in the background, and mobile applications depend on user interaction with a screen. These differences may shape acceptance in distinct ways.

#### 3.2.1 User-related factors

Demographic factors influencing older adults’ acceptance of health monitoring tools mainly include age, gender, educational attainment, and age-related health problems. Regarding age, Camp et al. (51) showed that older adults of advanced age have lower acceptance of environmental monitoring devices, yet they are willing to accept wearable devices, possibly because wearable devices offer more functions and are easier to operate. This suggests that age-related declines in sensory and cognitive function may reduce the perceived usability of some health monitoring tools. Regarding gender, Cavallo et al. (52) reported that older adults of different genders exhibit different levels of acceptance; male older adults place greater trust in health monitoring technology and may be more likely to accept and use it. In contrast, Deguchi et al. (53) reported the opposite finding, showing that women living alone had higher acceptance of health monitoring tools. This inconsistency may reflect differences in living arrangements: women living alone may have stronger health concerns and thus greater motivation to adopt monitoring tools, whereas men may rely more on technology trust. Regarding educational attainment, Chalkioti and Exarchos (83) indicated that older adults with lower educational attainment had lower acceptance of health monitoring tools, as their poorer comprehension and learning ability regarding new technologies and tools make it difficult for them to master the use of such tools. Simmich et al. (54) found that older adults tend to have poor digital skills and often interrupt their use of health monitoring tools due to operational errors, which may further undermine the usability of these tools. Together, these findings suggest that educational attainment influences acceptance not only through comprehension but also through digital skill development. Regarding age-related health problems, Kadylak and Cotten (55) demonstrated that poor health status in older adults limits their ability to access and communicate health information via technology, which may contribute to their inability or reluctance to use health monitoring tools. Additionally, Doyle et al. (56) showed that using health monitoring tools is particularly difficult for older adults with complex multimorbidity. Overall, demographic and health-related factors influenced acceptance in complex and sometimes contradictory ways, with gender effects varying by living arrangement and tool type. These findings suggest that demographic characteristics should not be treated as isolated variables but as interrelated factors shaped by social context.

Older adults face multiple psychological barriers when adopting health monitoring tools, including anxiety about and resistance to new technologies, a sense of burden when using the devices, and concerns about stigmatization and side effects. Regarding anxiety and fear toward new technologies, Herkert et al. (57) demonstrated that many older adults may experience discomfort, anxiety, and even fear toward new technologies when using health monitoring tools, perceiving considerable difficulties in using such technologies for health monitoring. Similar to the findings of Abdul Rahman et al. (58), older adults who have never been exposed to or used health monitoring tools lack confidence in using advanced technology. Regarding psychological burden and stigmatization, the sense of burden older adults feel when using health monitoring tools is also an important factor hindering acceptance. A study on fall detection devices (59) showed that large health monitoring tools may cause older adults to feel embarrassed during use, and the discreetness of the tool was found to be more important than its functionality. Older adults also expressed concerns about the potential stigmatization associated with using health monitoring tools specifically designed to monitor or assist older adults (60). A study (84) pointed out that health monitoring tools designed for the general public, rather than exclusively for older adults, can help improve their quality of life and health status, which may increase their acceptance of health monitoring. Regarding concerns about side effects, acceptance is also significantly influenced by worries about potential adverse effects of health monitoring tools; older adults may resist potential side effects such as allergic reactions and pain caused by implanted sensors (85). Meanwhile, the lack of validated measures of effectiveness for some health monitoring tools may lead to distrust and gradual discontinuation of use among older adults. Overall, psychological characteristics were consistently reported across studies, although the specific concerns varied—ranging from anxiety and lack of confidence to stigmatization and side effects. Notably, psychological factors were not uniformly negative: health anxiety and concerns about living alone also motivated some older adults, particularly women living alone, to adopt monitoring tools. This suggests that psychological factors can function as both barriers and facilitators depending on the individual’s circumstances.

#### 3.2.2 Tool- and context-level factors

The privacy and security of monitoring data are critical factors influencing older adults’ acceptance of health monitoring tools. Zhang et al. (86) demonstrated that due to a sense of domestic boundaries, safety concerns, and personal living habits, health monitoring tools encounter a “two-door problem”—difficulty gaining entry into both homes and communities. Older adults and their families often refuse to install smart devices equipped with image, sound, or pressure monitoring functions, fearing risks such as personal data leakage and property loss. These concerns reflect not only privacy but also a sense of control over one’s living environment. Comiskey et al. (61) used smart home monitoring devices to monitor daily home care for older adults and found that detecting only shadows, outlines, and coarse movements via cameras can effectively reduce the sense of surveillance intrusiveness; however, this approach limits the ability to recognize special situations such as falls or disease onset through specific movements or expressions, which may compromise the effectiveness of the tools. This finding highlights a trade-off between privacy protection and monitoring effectiveness. Furthermore, Drira et al. (87) indicated that control over the scope of information sharing is very important; some older adults are unwilling to share health monitoring data with family members or healthcare providers. Thus, privacy concerns extend beyond device installation to the sharing of data. Kim and Choi (62) showed that older women have higher privacy requirements than older men and tend to be more reserved when sharing personal health information. Overall, privacy and security concerns were consistently identified as barriers, with the tension between data protection and tool effectiveness emerging as a recurring theme. Privacy concerns varied across subgroups, indicating that privacy is not a binary concern but a matter of selective control over information.

Studies (63, 64) have shown that most older adults prefer interactive programs over one-way information delivery systems; factors such as small font size and poor text readability in user interfaces can all lead to difficulties in their interaction with applications. Portz et al. (65) demonstrated that older adults often find health monitoring tools difficult to use due to a lack of concise instructional prompts in the user interface. Frishammar et al. (66) showed that when health monitoring tools malfunction, a lack of timely operational guidance often leaves older adults feeling helpless and increases their usage burden, which may hinder their acceptance of these tools. Meanwhile, Yang et al. (41) pointed out that providing interactive informational support, personalized health features, and ensuring innovative, unique functionalities are important factors for improving acceptance of health monitoring tools. Furthermore, Haghi et al. (88) indicated that smart environmental monitoring is more user-friendly than wearable devices; for example, it can operate continuously 24 h a day with a long battery life, eliminating the need for older adults to recharge the system regularly, which may make it easier for them to accept and use. This suggests that the type of technology itself may shape the user experience, with ambient systems requiring less active user involvement. However, most age-friendly modifications of digital products remain very limited, merely reflected in larger fonts and icons and the removal of additional features from standard versions, with little substantial difference from ordinary versions (67). Overall, interaction experience emerged as a key determinant of acceptance, with older adults consistently favoring interactive, personalized, and low-burden interfaces. However, current age-friendly designs often remain superficial and do not fully address age-related cognitive and sensory declines.

The acceptance and use of health monitoring tools by older adults may be influenced by device appearance and invasiveness. Favorable aesthetic design and discreetness of system components may enhance older adults’ acceptance of these tools. Research (89) has shown that, in the home environment, minimizing device size can effectively reduce invasiveness, which may avoid discomfort for both users and visitors. This suggests that physical obtrusiveness is closely linked to perceived intrusiveness. Wu et al. (68) demonstrated that the major barrier to acceptance among older adults is the stigma associated with the devices, as large smart equipment such as robots, sensors, or other health monitoring devices tends to attract more attention from others. Thus, beyond physical discomfort, the social visibility of devices can also undermine acceptance. Avella-Rodriguez et al. (59) reported that, regarding preferences for wearable health monitoring tools, older adults tend to prefer wearing them on the wrist, while older women expect design elements such as jewelry to be incorporated. Overall, device appearance and invasiveness were consistently noted as important, with gender differences in design preferences and discreteness repeatedly emphasized. These findings suggest that the social meaning of a device—how visible it is and what it signals to others—may be as important as its technical performance in shaping acceptance.

Health monitoring tools generally entail high costs and prices due to the demands of research, design, and testing of advanced technologies such as high-precision sensors and data processing chips, as well as factors including brand premiums and market positioning. This limits older adults’ acceptance and use of such tools, and the challenge is particularly severe for older populations in rural areas, developing countries, and economically underdeveloped, low-income regions (69, 70). Furthermore, Tanaka et al. (71) showed that difficulty in technology access is one of the major barriers for older adults to use health monitoring tools. Thus, affordability and access are distinct but interrelated obstacles. As companies reduce labor and material costs, some health monitoring tools lack adequate technical support and after-sales service, which may also mislead users’ health monitoring. Dai and Lin (9) demonstrated that 70% of older adults believe that health monitoring tools require after-sales training services. Cumulatively, economic cost was consistently identified as a barrier across studies, particularly for older adults in rural and low-income regions, and inadequate after-sales support further compounds this challenge. Thus, economic barriers operated through two distinct pathways: affordability (whether older adults could pay for the tools) and access (whether the tools and supporting services were available to them). Both pathways disproportionately affected older adults in rural and low-income settings.

## 4 Discussion

### 4.1 Interpretation of findings

This review synthesized the literature on the factors influencing older adults’ acceptance of health monitoring tools. The findings indicate that acceptance is shaped by a complex interplay of user-related factors (demographic, health-related, and psychological characteristics) and tool- and context-level factors (interaction experience, device appearance, privacy and security, and economic cost), and that current acceptance models identified in this review—though diverse—have yet to fully capture the needs of older adults across diverse populations.

At the individual level, the review found that psychological factors can act both as barriers and as intrinsic drivers (90). The health anxiety caused by living alone may be associated with older women’s interest in self-management of health and with higher acceptance of and dependence on smart wearable technologies (53). This bidirectional nature suggests that psychological characteristics are not fixed traits but can be reshaped through targeted support (72, 73). These psychological characteristics are often compounded by limited educational attainment and digital literacy. Lower educational attainment (83) and poor digital skills (54) were associated with greater usage difficulties, and older adults with these characteristics were more likely to discontinue use when encountering operational errors.

For older adults with complex health conditions, the review found that existing tools struggle to address multimorbidity (56, 74). Cimperman et al. (75) showed that healthcare professionals’ recommendations of telehealth devices may enhance perceived usefulness and thereby increase older adults’ acceptance. This highlights the important role of professional endorsement in facilitating acceptance among this subgroup.

Beyond individual and health-related factors, the design of the tools themselves and the broader context play a critical role. In the context of the digital divide, privacy and security throughout the collection, storage, transmission, and use of older adults’ health data are prerequisites for widespread acceptance (91, 92). As noted in the Results, concerns about personal data leakage and property loss often lead older adults and their families to refuse smart devices with image or sound monitoring functions (86). Control over the scope of information sharing is particularly important for older adults (87), and older women are more sensitive to the potential risks of personal health data and privacy breaches, with a stronger need for psychological security and privacy protection (62). Opaque algorithms and inadequate security measures can reduce users’ trust and reliance on devices (93). These concerns are not merely technical but reflect a deeper need for control and dignity in later life.

Acceptance drivers differed across tool types: wearable devices were more accepted when discreet, wrist-worn, and aesthetically appealing; smart home and ambient systems were favored for passive, continuous operation with less user involvement; mobile health applications depended heavily on interface design and digital skills; and assistive robots raised stronger concerns about stigmatization and social visibility. These differences indicate that a one-size-fits-all approach is unlikely to be effective, and that design and intervention strategies should be tailored to specific tool types.

Health monitoring tools generally entail high costs and prices due to the research and development expenses of high-precision sensors and data processing chips, as well as factors including brand premiums and market positioning (94–96). This challenge is particularly severe for older populations in rural areas, developing countries, and economically underdeveloped regions (78). Difficulty in technology access has become one of the major barriers for older adults to use health monitoring tools (79), and some products, due to cost reduction, lack adequate technical support and after-sales service, which may also mislead users’ health monitoring (97). Beyond cost alone, the digital divide—encompassing disparities in healthcare access, differences in economic conditions, and gaps in digital literacy and educational attainment—collectively shapes the differentiated acceptance patterns of health monitoring tools among older adults (98, 99).

Finally, at the theoretical level, although numerous acceptance models exist, among the models identified in this review, no model specifically addressing the acceptance of health monitoring tools among older adults has yet gained broad social recognition. For example, the Senior Technology Acceptance Model treats individual characteristics or external environment as exogenous variables, with insufficient analysis of product design elements and older adults’ needs, thus offering limited practical guidance for improving health monitoring tools (100). Other theoretical models also have limitations in this context. Driven by population aging and societal demand, the geriatric care and smart health industries have grown rapidly, with a proliferation of smart health products and strong public interest in health monitoring and promotion tools (101, 102). However, medical and health research, which plays a central role in health promotion, has lagged behind this trend. Theoretical and applied research on such products remains underdeveloped in many aging societies.

### 4.2 Implications and recommendations

Based on the evidence reviewed, the following recommendations are proposed.

Society and the geriatric care industry need to fully recognize the factors influencing the acceptance of health monitoring tools and implement targeted interventions on modifiable factors, which may enhance their acceptance, utilization, and penetration rates among older adults (103, 104).

The prerequisite for enhancing older adults’ acceptance of health monitoring tools lies in fully understanding their cognitive characteristics and actual usage difficulties, and providing support systems matched to their abilities (105). Accordingly, the key lies in providing targeted health education and psychological support. Through product promotion and targeted guidance, older adults should build confidence in health monitoring tools and recognize their value in early detection and prevention of disease deterioration.

From a design perspective, some countries have introduced age-friendly digital technology policies that emphasize optimizing user experience for older adults. China’s Work Plan for Promoting the High-Quality Development of Age-Friendly Digital Technology (106) provides one example of such guidance. Developers should accommodate age-related vision and hearing impairments and cognitive decline by providing clear voice prompts, streamlined one-touch monitoring functions, one-click customer service for real-time assistance, and weekly or monthly health reports presented with charts. For older adults with low education or poor digital skills, manufacturers and vendors should organize public training sessions and develop intuitive visual guides.

In clinical practice, for complex cases such as multimorbidity, health monitoring tools should collaborate with medical institutions to enable real-time monitoring and timely intervention via remote monitoring (107). Hospitals could provide health monitoring advice, such as identifying key indicators requiring continuous attention (e.g., blood pressure, blood glucose, heart rate), setting regular monitoring times, tracking fluctuations, and integrating monitoring into daily routines (76).

Within the family context, family members should assist older adults in using the tools; specifically, family members can pair the devices with their own smart devices, help configure settings or medication reminders, discuss results, and interpret ambiguous recommendations, thus promoting acceptance of new technologies (108).

At the tool design level, when developing products targeted at older adults, it is essential to gain an in-depth understanding of their characteristics and genuine needs, ensure a simple interface and intuitive operation, and automate tasks as much as possible to reduce user interaction burden and improve satisfaction (77, 109). Product design should consider psychological burden by reducing device size, refining aesthetics within medical device regulations, and incorporating jewelry-like elements to reduce stigma (110).

Regarding privacy protection, it is recommended that older adults be allowed to customize monitoring items and frequency and decide whether to share results with family or healthcare providers. For sensitive indicators such as psychological or specialty examinations, anonymous identifiers could replace personal information to enhance trust.

At the policy level, reducing prices while providing after-sales service commensurate with product value may help reduce older adults’ perceived risk and improve their perceived value of health monitoring tools. Addressing these economic barriers requires systemic policy interventions. In line with the growing attention to the silver economy in many aging societies (111), dedicated financial subsidies should be established with multi-tiered standards. Full or high-percentage subsidies should be provided for older adults living alone, empty-nesters, and those with disabilities, covering basic health monitoring tools such as smart blood pressure monitors, glucometers, heart rate monitoring wristbands, and pulse oximeters. Subsidy procedures should be simplified, with funds either directly distributed to older adults or used to offset purchase costs through partner merchants, which may encourage and support older adults to purchase and use health monitoring tools for self-management.

Finally, at the theoretical level, the authors recommend that healthcare professionals pay greater attention to the geriatric care and smart health industries, strengthen theoretical and applied research, and develop innovative models suited to older adults or adapt existing models to local aging contexts, which may increase research output, empower industry development, and better fulfill the responsibility of safeguarding human health.

In summary, enhancing older adults’ acceptance of health monitoring tools requires coordinated efforts across multiple dimensions—addressing individual cognitive and psychological characteristics, optimizing tool design and privacy safeguards, bridging economic and digital divides through policy support, and advancing theoretical models tailored to diverse aging contexts.

## 5 Strengths and limitations

Several limitations of this review should be acknowledged. First, the literature was identified through a snowballing approach, drawing primarily on English and Chinese literature; this may have resulted in the omission of relevant studies published in other languages or indexed in databases not covered. Second, the heterogeneity of the included studies—in terms of study design, sample characteristics, measurement tools, and outcome indicators—limited the comparability of findings. No formal risk-of-bias assessment was conducted, and no quantitative synthesis was performed, consistent with the narrative nature of this review. The majority of the included studies were cross-sectional, which makes it difficult to establish causal relationships between the identified influencing factors and older adults’ acceptance. Furthermore, a considerable proportion of the studies relied on self-reported measures and convenience sampling, which may introduce response bias and limit the generalizability of the findings. In addition, the included studies were predominantly from high-income and upper-middle-income countries, and research from low- and middle-income countries remains limited. Third, as a narrative review, the synthesis and interpretation of findings inevitably involved a degree of subjective judgment. Fourth, given the rapid pace of technological advancement in geriatric care and smart health, some findings may become outdated as new tools and features continue to emerge. Despite these limitations, this review contributes to a more coherent understanding of the factors influencing older adults’ acceptance of health monitoring tools.

## 6 Future research directions

Future research should address the above limitations and extend the current evidence base in several directions. First, longitudinal and prospective studies are urgently needed to examine how older adults’ acceptance of health monitoring tools evolves over time. Current studies are limited in their ability to capture the dynamic changes in acceptance during the process of technology adoption and sustained use. Second, future research should flexibly apply and validate existing theoretical acceptance models. In particular, STAM, which has been shown to be effective in technology acceptance research among older adults, warrants further exploration in the context of health monitoring tools, as it was not applied in the studies identified in this review. Moreover, researchers should endeavor to develop innovative models suited to older adults or adapt existing models to diverse aging contexts, taking into full account the unique sociocultural environment, family structures, and policy frameworks. Although this review identified studies from diverse regions, research from low- and middle-income countries remains limited. Given the distinct cultural, economic, and social contexts of these settings, and their rapidly aging populations, further studies conducted in these regions would substantially enrich the current understanding.

## 7 Conclusion

This review synthesized the literature on older adults’ acceptance of health monitoring tools. Overall, acceptance is shaped by a complex interplay of user-related factors—including demographic and health-related characteristics and psychological characteristics—and tool- and context-level factors, such as privacy and security, interaction experience and ease of use, device appearance and invasiveness, and economic cost. Among the theoretical models identified in this review, TAM and UTAUT are the most widely applied, whereas no study included in this review applied STAM to health monitoring tool acceptance, representing an underrepresented area within the identified literature.

Older adults’ attitudes toward health monitoring tools remain generally positive despite the identified barriers. When concerns regarding privacy, ease of use, and cost are adequately addressed, older adults’ acceptance of health monitoring tools may be improved.

Future research should explore how older adults’ acceptance of health monitoring tools interacts with educational level, technology familiarity, and strategies aimed at improving user experience. Both the development of innovative theoretical models suited to diverse aging contexts and longitudinal studies examining acceptance over time are warranted to deepen understanding of this evolving field. Ultimately, building an age-friendly and technologically inclusive smart health ecosystem is essential to empower older adults to benefit from advances in health monitoring technologies.
